# *Thesiumnautimontanum*, a new species of Thesiaceae (Santalales) from South Africa

**DOI:** 10.3897/phytokeys.109.28607

**Published:** 2018-10-02

**Authors:** Miguel Angel García, Daniel L. Nickrent, Ladislav Mucina

**Affiliations:** 1 Real Jardín Botánico CSIC, Plaza de Murillo 2, 28014 Madrid, Spain Real Jardín Botánico Madrid Spain; 2 Royal Botanic Gardens Kew, Richmond, Surrey, TW9 3AE, UK Royal Botanic Gardens Surrey United Kingdom; 3 Department of Plant Biology, Southern Illinois University, Carbondale, IL 62901-6509, USA Southern Illinois University Carbondale United States of America; 4 School of Biological Sciences, The University of Western Australia (M090), 35 Stirling Highway, WA 6009 Crawley, Australia The University of Western Australia Crawley Australia; 5 Department of Geography and Environmental Sciences, Stellenbosch University, Private Bag X1, Matieland 7602, South Africa Stellenbosch University Matieland South Africa

**Keywords:** Capensis, endemic plant, Matroosberg mountain, parasitic plant, Santalaceae

## Abstract

*Thesiumnautimontanum* M.A. García, Nickrent & Mucina, a new species from the Matroosberg Mt. of Western Cape Province of South Africa, is described and illustrated. This species shows several morphological features unusual for the genus including stem sympodial branching, indeterminate spicate inflorescences subtended by numerous bracts and fleshy, non-trichome tissue lining the inside of the corolla lobes. Molecular phylogenetic analyses using nuclear ribosomal internal transcribed spacer sequences place this taxon as sister to all African, Madagascan and South American *Thesium* species. Given that only two proximal populations are known, this species is of conservation concern.

## Introduction

*Thesium* L. is the most speciose genus in the sandalwood order (Santalales) with over 350 species (Nickrent and García 2015). The genus has traditionally been placed in Santalaceae s. lat., however, the Nickrent et al. (2010) classification places *Thesium* in Thesiaceae. Recent taxonomic changes have expanded the genus by including previously described genera such as *Kunkeliella* Stearn and *Thesidium* Sond. (Forest and Manning 2013). Although *Thesium* can be found in Europe, Asia, Australia, South America and North America (by human introduction), the vast majority of species are African with the greatest diversity being found in the Cape region. Indeed, the fact that *Lacomucinaea* Nickrent & M.A. García and *Osyridicarpos* A. DC. are sister to *Thesium* (Nickrent and García 2015) and are both restricted to southern Africa indicates this is the likely region where the genus originated.

The Fynbos Biome, embedded within the South African Cape Floristic Region, sometimes referred to as a Floristic Kingdom (Takhtajan 1986), is one of the most floristically diverse regions in the world (Mucina and Rutherford 2006). Indeed, of the ca. 170 named species of *Thesium* from South Africa, over 40 of these are found only in the Cape region. High diversity complicates identification of all angiosperms but this is particularly true in poorly documented genera such as *Thesium*. The last major taxonomic works dealing with *Thesium* in South Africa were by Hill (1915, 1925), but since then a number of new species have been named. The keys presented in Hill’s works are today not particularly useful in identifying South African *Thesium* because they do not include more recently named species, e.g. those from Brown (1932) or Compton (1931). Moreover, the characters used to distinguish species are not effective when a broad spectrum of samples is examined. This is exacerbated by phenotypic plasticity seen in some species across developmental stages and under different ecological conditions (fire, grazing, elevation, substrate etc.).

This paper describes a new species of *Thesium* that is exceptional from several perspectives. In terms of morphology, it shows several features that are unique in the genus. From the molecular phylogeny perspective, this species holds a special place in that it is sister to the entire clade of African *Thesium* (250 species).

## Materials and methods

The morphology of this new species was studied from herbarium samples collected by J. Šibík and I. Šibíková in October 2007 and Mr. Eugene Pienaar in December of the same year. Material from the herbarium samples was rehydrated and examined with a stereomicroscope. DNA extraction, PCR and sequencing of nuclear internal transcribed spacer of ribosomal DNA (ITS) was conducted as specified in Nickrent and García (2015). The new ITS sequence generated for this study is available in GenBank with accession number MH807489.

The ITS sequence from the new species was added to the alignment from Nickrent and García (2015). This alignment has been submitted to TreeBase with study reference number S23256. Phylogenetic analyses were conducted as detailed in Nickrent and García (2015) and included maximum likelihood (ML), maximum parsimony (MP) and Bayesian inference (BI) analyses.

**Figure 1.**
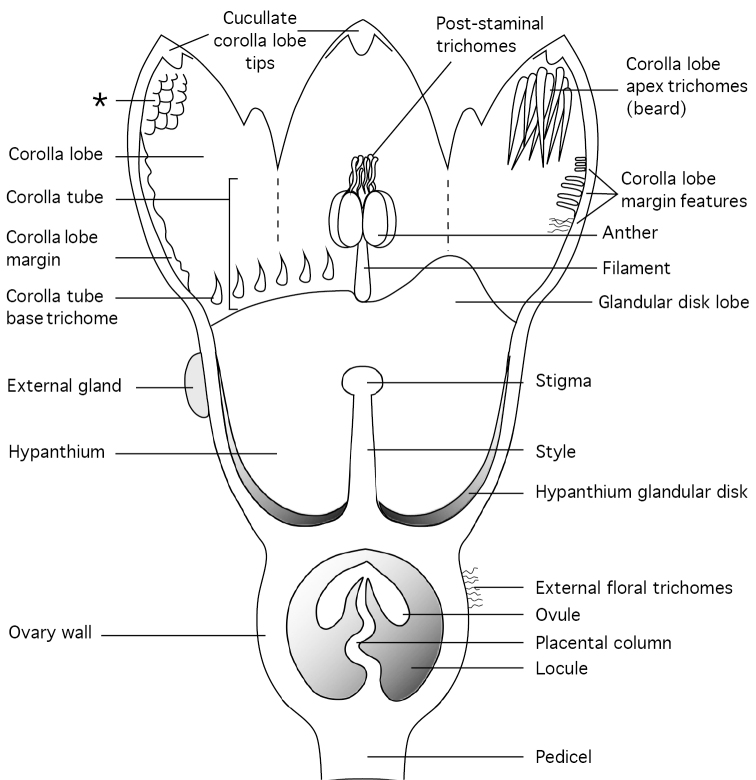
Diagramatic longitudinal section through a generalised *Thesium* flower. The figure was composited from numerous species, hence not all features occur in one species. Nearly all characters shown are variable within the genus. The asterisk (*) indicates the unique fleshy tissue seen lining the corolla lobes in *T.nautimontanum*.

To aid interpretation of the flower description, a generalised Thesium flower longitudinal section is shown in Fig. 1.

The extent of occurrence (EOO) and area of occupancy (AOO) were determined using the Geospatial Conservation Assessment Tool with a default cell width of 2 km (GeoCAT, Bachman et al. 2011). The conservation status of the species was determined by consulting the Guidelines for Using the IUCN (International Union for Conservation of Nature) Red List Categories and Criteria (IUCN 2014).

## Taxonomy

### Thesium nautimontanum

Taxon classificationPlantaeSantalalesThesiaceae

M.A. García, Nickrent & Mucina sp. nov.

urn:lsid:ipni.org:names:77190706-1

Figs 2 , 3

#### Type.

SOUTH AFRICA. Western Cape Province, Cape Winelands District, Matroosberg, southern slope, 33°23'49.3"S, 19°40'29.5"E, elev. 1016 m; 6 Oct 2007, *J. Šibík & I. Šibíková MS463* (holotype: MA-877227).

#### Diagnosis.

Squamate, glabrous, perennial herb 30–35 cm tall with erect, terete to ribbed sympodial branches; leaves reduced to triangular, acute scales up to 1.7 mm long, increasing in size and density apically, grading into inflorescence bracts. Inflorescences terminal, oblong, crowded spikes bearing up to 50 bracteate flowers, ending in a group of sterile bracts. Flower ca. 2.0 mm long, greenish outside, whitish inside, with triangular external glands between the corolla lobes, internal surface of lobes bearing thick, irregular, spongy and somewhat papillose tissue. Fruit wall marked with conspicuous, strongly raised reticulations.

#### Description.

Perennial herb, squamate, all parts glabrous; rootstock slender, bearing one or few erect flowering stems 30–35 cm tall with sympodial branching that occurs mostly at the base of inflorescences, branches erect, terete or shallowly ribbed. Leaves all reduced to minute scales, the lower ones 0.5–1.0 × 0.8–1.7 mm, broadly triangular, acute, with darkened tips, erect and more or less appressed to the stem, increasing in size upwards and transitioning into bracts, 1.1–1.9 × 0.8–1.1 mm, ovate-triangular to ovate, acute to acuminate, with entire margins, incurved to naviculate but lacking a marked midrib and bearing in their axils undeveloped buds, progressively more crowded towards the base of the inflorescence, greenish to straw-coloured. Inflorescences terminal (on long and short shoots), oblong, spikes 10.5 × 18.5 mm, bearing (15-)24–50 crowded flowers, ending in a group of about 7–14 sterile bracts. Flowers ca. 2.0 mm long, solitary and sessile in the axil of each bract or with a short (up to 0.8 mm long) pedicel. Flower bracts 1.5–2.2 × 1.0–1.4 mm, similar in shape to the bracts at the base of the inflorescence; bracteoles 2, similar to bracts but smaller. Corolla greenish outside, whitish inside; external glands between the corolla lobes inconspicuous, triangular-ovate, pale orange in colour; hypanthium shallow, 0.2 mm long, internal surface glandular; corolla tube ca. 0.4 mm long; corolla lobes 0.7–0.8 × 0.6–0.7 mm, ovate or ovate-triangular, ± obtuse to slightly hooded and with inflexed tips on fruit, erect, margins entire, lacking hairs inside but with a thick, irregular, fleshy and somewhat papillose tissue. Staminal filaments inserted at junction of hypanthium and corolla tube, very short and inconspicuous, stamen not reaching the corolla lobe sinuses, anthers rounded ca. 0.3 mm in diameter, attached to the corolla tube by a small tuft of hairs. Style very short (ca. 0.1 mm) or lacking, thus stigma sessile, lobes not apparent, reaching the base of the anthers. Placenta twisted, ovules 3. Fruits ca. 3.0 mm long, ovary portion subglobose, 1.5–2.1 × 1.6–2.0 mm, greenish, with slightly marked ribs but reticulations strongly raised, crowned with the 1.0 mm long corolla remnant, pedicel not enlarging into an elaiosome.

**Figure 2.**
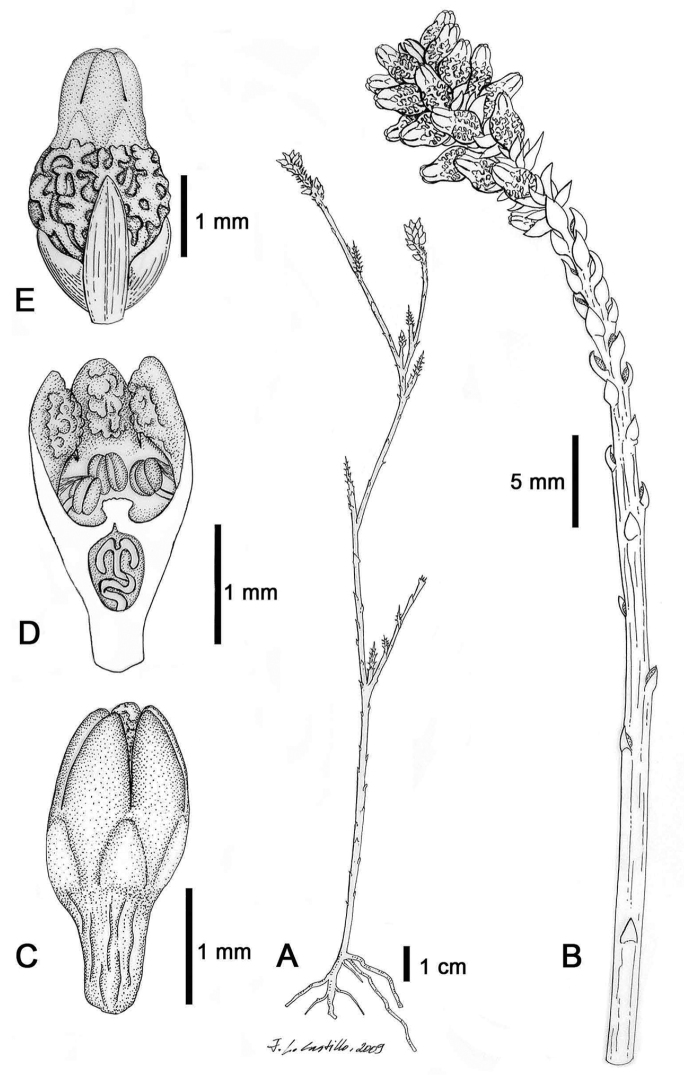
*Thesiumnautimontanum* M.A. García, Nickrent & Mucina, sp. nov. **A** habit of plant showing sympodial branching **B** flowering shoot showing squamate condition, vegetative bracts grading into floral bracts and terminal spicate inflorescence **C** flower, lateral view **D** flower longitudinal section **E** young fruit subtended by bract and two bracteoles. Drawing by J. L. Castillo.

**Figure 3.**
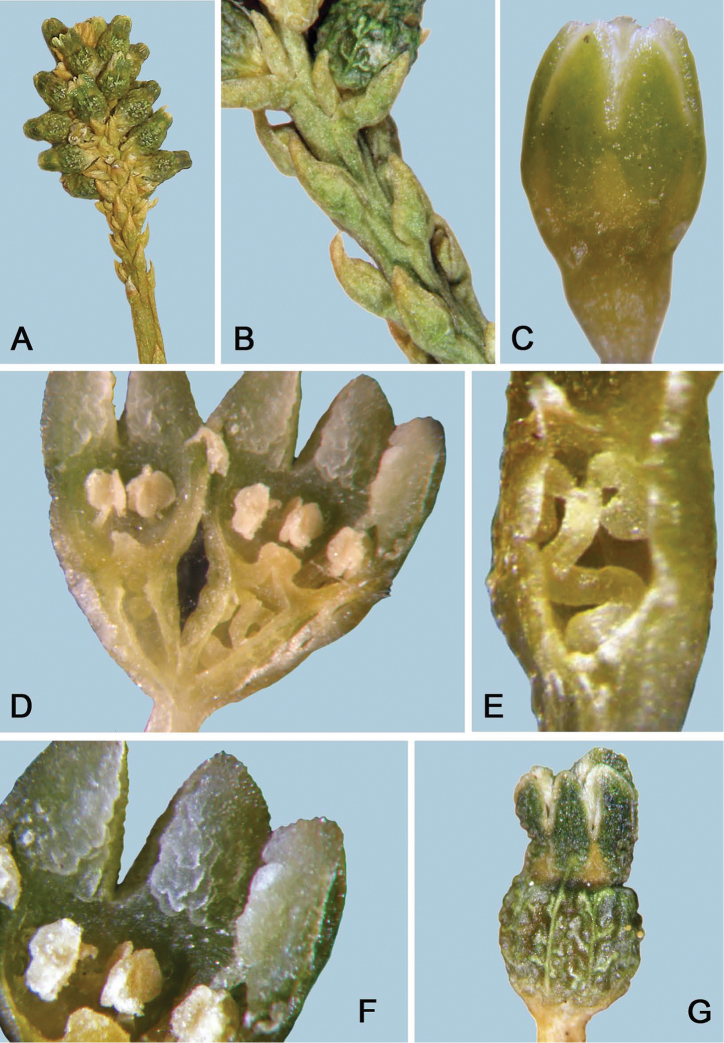
*Thesiumnautimontanum* from the type herbarium specimen. For dimensions, see Figure 2. **A** spicate inflorescence subtended by bracts **B** close-up of inflorescence bracts **C** flower, lateral view, showing triangular external glands in corolla lobe sinuses **D** flower longitudinal section **E** ovary longitudinal section showing twisted placental column and ovules (one ovule removed to visualise the placentation) **F** close-up view of interior surface of corolla lobes showing unusual fleshy tissue on inner surface of corolla lobes **G** young fruit showing persistent corolla and reticulate outer wall of ovary.

#### Distribution and ecology.

Both collections of this species were made on the Matroosberg Mt. in the Southern Hex River mountain range, Western Cape Province, Cape Winelands District, northwest of the De Doorns settlement.

The holotype collection, from 1016 m elevation, occurred on the southern slopes of the Matroosberg Mt. in the FFs8 South Hex Sandstone Fynbos vegetation according to Rebelo et al. (2006). The soils are acidic lithosols derived from Ordovician sandstone of the Table Mountain Group. This vegetation is, on the type locality, characterised by sclerophyllous, microphyllous fynbos scrub dominated by proteaceous, ericaceous and asteraceous shrubs, with rich graminoid understory containing numerous Cyperaceae and Restionaceae. The area of the South Hex Sandstone Fynbos receives, on average ca. 950 mm of rain annually, peaking from May to August. Summer weather may include heavy mist precipitation at higher altitudes. The mean daily temperatures are 26°C and 2.7°C for February and July, respectively.

The second population was discovered at an elevation of 1920 m, close to the summit of Matroosberg (2249 m). Here *Thesiumnautimontanum* was found in a slight depression supporting a dense restioid-ericoid fynbos wetland (dominated by graminoids of the family Restionaceae and shrubs of *Erica* L.), surrounded by dry fynbos scrub of the FFs30 Western Altimontane Sandstone Fynbos. The soils here are nutrient-poor and sandy, overlying Table Mountain Sandstone. In contrast to the South Hex Sandstone Fynbos, the altimontane fynbos unit receives higher mean annual rainfall (1380 mm on average), peaking from May to August; frequent fog occurrence (due to summer tradewinds) may carry additional precipitation, mitigating the effect of summer drought at high elevations – a phenomenon common to many Cape Fold Mountains (see Rebelo et al. 2006, p. 69). The mean annual temperature is lower, as it ranges between 22.9°C (February) and 0.1°C (July). The summit regions of Matroosberg regularly receive snow during austral winter.

#### Phenology.

The specimens examined had spikes containing both flowers and maturing fruits and were thus at similar developmental stages. As the lower elevation collection was flowering in October and the higher elevation one in December, one can extrapolate that the flowering time ranges from ca. September to January.

#### Etymology.

The specific name stems from the Matroosberg Mt. where the species was first discovered. In Afrikaans, “matroos” means “sailor” and “berg” means mountain. As the Code of Botanical Nomenclature suggests, epithets formed from a geographic name should be in adjectival rather than substantive form, “nauta” (sailor) and “mons” (mountain) become *autimontanum*.

#### Conservation status.

According to the World Database of Key Biodiversity Areas (http://www.keybiodiversityareas.org/home), the Hex River Mountains are surrounded by globally and regionally significant areas (UNESCO World Heritage Site ‘Cape Floral Region’), but are themselves not currently included. Due to rugged terrain, nutrient-poor soils not viable for large-scale agricultural activities and relative low-key tourism interest (more of importance on the northern flank of the Matroosberg Mt.), the biota of the Hex River Mts. is not under direct, unmitigated threat. However, fynbos scrub is a naturally fire-prone ecosystem, yet too frequent (or too hot) fires might have negative impacts on the natural post-fire regeneration (e.g. Le Maitre and Midgley (1992), Bond and van Wilgen (1996)). The morphology of Pienaar’s collections, having a loose bunch of thin roots, suggests the species is a seeder, hence regenerates after fire from the seed bank.

A section of the Hex River Mts. is under management of CapeNature (the Western Cape provincial nature conservancy authority) and is formally protected in Bokkeriviere Nature Reserve. The coordinates of E. Pienaar M1249 are at 1920 m a.s.l., which place this higher elevation population just south of the boundary of the Reserve. This may well indicate that the species might also occur within the boundaries of the Reserve.

The limited geographic distribution of *Thesiumnautimontanum* is, however, of concern. Using GeoCAT for the two populations, the extent of occurrence was 0.0 and the area of occupancy was estimated at 8.0 km^2^ which translates to an IUCN Red List category of Critically Endangered, criteria B2 (IUCN 2014).

Further exploration of these regions should be conducted to determine whether additional populations exist. Even if more populations are encountered, most likely it will stay in the category of Critically Endangered because it is an endemic species restricted to the Matroosberg Mt.

#### Additional specimens examined.

SOUTH AFRICA. Western Cape Province, Cape Winelands District, Matroosberg, southern slope, 33°23'43.48"S, 19°40'19.46"E, alt. 1920 m, 7 Dec 2007, *E. Pienaar M1249* (MA-877228, NBG).

#### Phylogenetic analyses.

**Figure 4.**
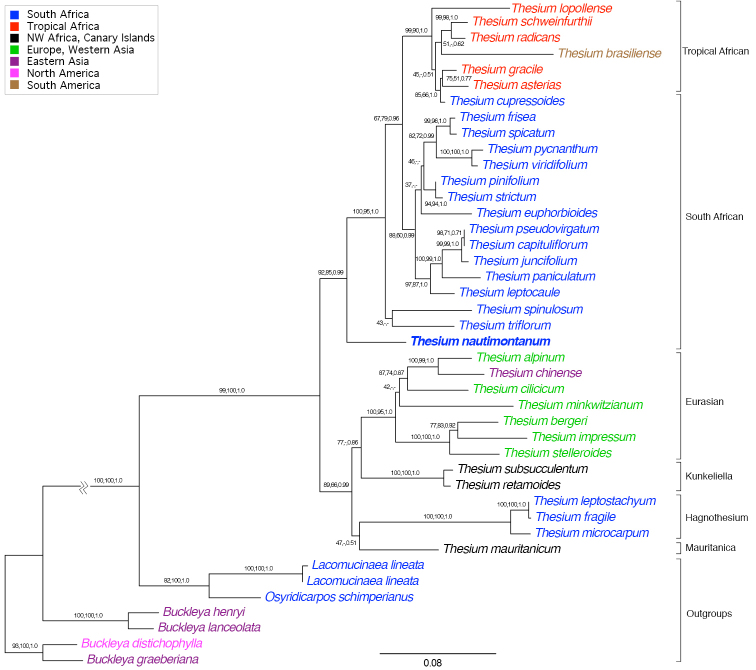
Phylogram for Thesiaceae that includes *Thesiumnautimontanum* sp. nov. (bold). Values at the nodes indicate maximum likelihood bootstrap, maximum parsimony bootstrap and Bayesian posterior probabilities. The scale bar represents number of substitutions per site. Branch lengths are proportional to the amount of substitutional change, except for the branch connecting the outgroup to the ingroup (here shorted for graphical purposes). Group (clade) names are from Nickrent and García (2015). The colour coding for the taxa indicates the general geographic distribution for the species as shown in the inset.

The molecular phylogenetic analyses of the ITS data using the three inference methods (ML, MP and BI) produced results generally comparable to those reported in Nickrent and García (2015), but in addition strongly supported *Thesiumnautimontanum* as sister to the South African and Tropical African clades (Fig. 4).

## Discussion

As far as known, *Thesiumnautimontanum* is restricted to the Matroosberg Mt., the highest mountain of the Hex River mountain range. This geomorphologically diverse landscape supports many local and regional endemic plants (see descriptions of the relevant vegetation types in Rebelo et al. 2006). Indeed, two other species of *Thesium*, *T.annulatum* A.W. Hill and *T.microcephalum* A.W. Hill, are endemic to the mountain range as well. Discovering a new species of *Thesium* in South Africa is not in itself remarkable, however, *T.nautimontanum* has particular importance for several reasons. According to our review of morphological characters from descriptions, type material and herbarium and living specimens from species across the entire area of distribution of the genus, it has a combination of features not seen in any other *Thesium* species. The inside surfaces of the corolla lobes are covered with spongy, irregular lumps of tissue that defy description. This feature is neither the hairs seen in Sect. Barbata A.W. Hill, nor the papillae or short hairs seen along the corolla lobe margins in other species (e.g. Subsect. Fimbriata A.W. Hill). The manner of inflorescence and branch development is also unique in *Thesium*. The bracts at the base of the spikes bear undeveloped buds that start forming some flowers when the upper ones have developed fruits. Each bud forms a shoot that will generate another inflorescence and so on, thus resulting in the characteristic sympodial branching pattern of this species. Another uncommon feature in the inflorescence is the presence of sterile bracts at the apex. The inflorescence appears to be truly indeterminate because there is not a terminal flower. In many species of *Thesium* (e.g. *T.aggregatum* A.W. Hill, *T.capitatum* L., *T.diversifolium* Sond., *T.scabrum* L. and *T.spicatum* L.), the inflorescence appears to be a spike, but upon further inspection, it can be seen that it is cymose (monotelic) and determinate with the main and lateral axes terminating in flowers. A few species of *Thesium*, like *T.impeditum* A.W. Hill or *T.gnidiaceum* A. DC., have indeterminate vegetative growth from the apical part of the inflorescence. This can later generate another inflorescence, having two (or more) inflorescences on the same axis separated by leafy segments. This feature is lacking in *T.nautimontanum*. Finally, the reticulations present on the fruit walls are also unusual. The 5 main longitudinal ribs are scarcely marked but the reticulations are strongly raised and swollen, giving a characteristic tuberculate surface.

The present molecular phylogenetic analyses are in agreement with another study with greater taxon sampling (390 accessions representing over 200 taxa) that is currently being prepared for publication (D. Nickrent et al., in prep.). Interestingly, using chloroplast spacer sequences, this species is sister to most African *Thesium*, however, the equivalent basalmost position is occupied by two clades, one containing *Thesiumspinosum* L.f. and *T.spinulosum* A. DC. and the next one composed of seven species that often bear simple or compound dichasial inflorescences that mostly occur in the South African Karoo. Incongruences between the nuclear and chloroplast gene trees are currently being investigated and preliminary data indicate chloroplast capture may be occurring, thus obscuring species relationships when using the plastid data.

## Supplementary Material

XML Treatment for Thesium nautimontanum
